# BAM8-22 and its receptor MRGPRX1 may attribute to cholestatic pruritus

**DOI:** 10.1038/s41598-019-47267-5

**Published:** 2019-07-26

**Authors:** Babina Sanjel, Han-Joo Maeng, Won-Sik Shim

**Affiliations:** 1 College of Pharmacy, Gachon University, Hambakmoero 191, Yeonsu-gu, Incheon 21936 Republic of Korea; 2 Gachon Institute of Pharmaceutical Sciences, Hambakmoero 191, Yeonsu-gu, Incheon 21936 Republic of Korea

**Keywords:** Sensory processing, Molecular neuroscience

## Abstract

Pruritus is an unexpected symptom observed in cholestasis and its mechanism is still unclear. Here, we show that bovine adrenal medulla (BAM) 8–22, an endogenous itch-inducing peptide, could be involved in cholestatic pruritus. It was found that bile duct ligation (BDL) mice, an obstructive cholestasis model, showed increased spontaneous scratching behaviour. Importantly, the mRNA level of proenkephalin, a precursor polypeptide of BAM8-22, was significantly increased in the skin of BDL mice. Furthermore, the mRNA level of *Mrgprx1*, which encodes a receptor for BAM8-22, was significantly increased in the dorsal root ganglia (DRG) of BDL mice. This was further confirmed by elevation of intracellular calcium levels upon BAM8-22 treatment in primarily-cultured DRG neurons. In addition, BDL mice showed augmented scratching behaviour by BAM8-22, indicating enhanced activity of MRGPRX1. Moreover, the skin homogenate of BDL mice induced elevation of intracellular calcium levels through MRGPRX1. Finally, among the various bile acids, chenodeoxycholic acid significantly increased proenkephalin transcription in a human keratinocyte cell line (HaCaT). In conclusion, cholestatic pruritus could be attributed in part to enhanced action of both BAM8-22 in the skin and its receptor MRGPRX1 in sensory neurons.

## Introduction

Cholestasis, characterized by decreased bile flow, occurs due to impaired bile secretion from hepatocytes or obstruction of intra- or extrahepatic bile ducts. Generally, cholestasis is accompanied by typical symptoms such as increased accumulation of bile acids in circulation, jaundice, dark urine, and steatorrhea. Surprisingly, one of the unexpected clinical indications of cholestasis is pruritus^1,2^. Although the cholestasis-associated pruritus has been known for years, the precise mechanism underlying pruritus is still not understood^3^. Previous studies indicate that bile acids are the main mediators of cholestasis-induced pruritus because of the nature of the disease^4–7^. In agreement with this notion, reports have shown that certain bile acids, such as deoxycholic acid (DCA), can induce scratching behaviour in mice via activation of a bile acid receptor GPBAR1 (G protein-coupled bile acid receptor 1, also known as TGR5) and an ion channel TRPA1 (Transient receptor potential cation channel subfamily A member 1) in primary sensory neurons^6,7^. Recently, it was further identified that MRGPRX4 (MAS-related GPR family member X4) is also activated by bile acids that may contribute to cholestatic pruritus^8^.

However, bile acids may not completely explain cholestatic pruritus^4^. Indeed, certain reports suggest that bile acids are not the major pruritogens of cholestasis. For instance, pruritus tends to disappear in patients of liver failure with high levels of bile acids^9^. In addition, not all patients with cholestasis reported pruritus, and pruritus often fluctuated, independent of the serum concentration of bile acids^4,9^. Furthermore, the amount of DCA in circulation diminishes with deterioration of cholestasis^10^. Therefore, these observations imply the existence of pruritogen(s) other than bile acids responsible for the cholestatic pruritus.

In the present study, we propose that bovine adrenal medulla (BAM) 8–22, an itch-inducing endogenous peptide derived from proenkephalin, may mediate cholestatic pruritus. Proenkephalin is an endogenous polypeptide precursor, which is converted into various types of polypeptides such as enkephalin, adrenorphin, amidorphin, peptide B/E/F, BAM18P, BAM20P, and BAM22P via proteolytic cleavage. BAM8-22 is a 15-amino acid cleavage product of BAM22P, which specifically activates its unique receptor, MRGPRX1 (MAS-related GPR family member X1, also known as MRGPRC11), and induces itch via the Gα_q/11_ pathway^11,12^. Additionally, activation of MRGPRX1 by BAM8-22 leads to further activation of TRPA1, a non-selective cation channel responsible for various histamine-independent itch pathways^13^.

Therefore, in the present study, we investigated changes in the expression of genes/proteins related to BAM8-22 under cholestasis conditions using an animal model of extrahepatic biliary obstruction of common bile duct ligation or BDL^14–16^. We observed that enhanced action of BAM8-22, as well as the expression and function of MRGPRX1, were significantly increased in BDL mice. In addition, BDL mice showed enhanced spontaneous scratching behaviour and increased sensitivity to BAM8-22. Therefore, the present study strongly implies that BAM8-22 may play a major role in chronic itching observed under cholestasis conditions and indicates a possible new target for developing therapeutics against cholestasis-related pruritus.

## Results

### Induction of cholestasis by bile duct obstruction increased spontaneous scratching in mice

**Figure 1:**
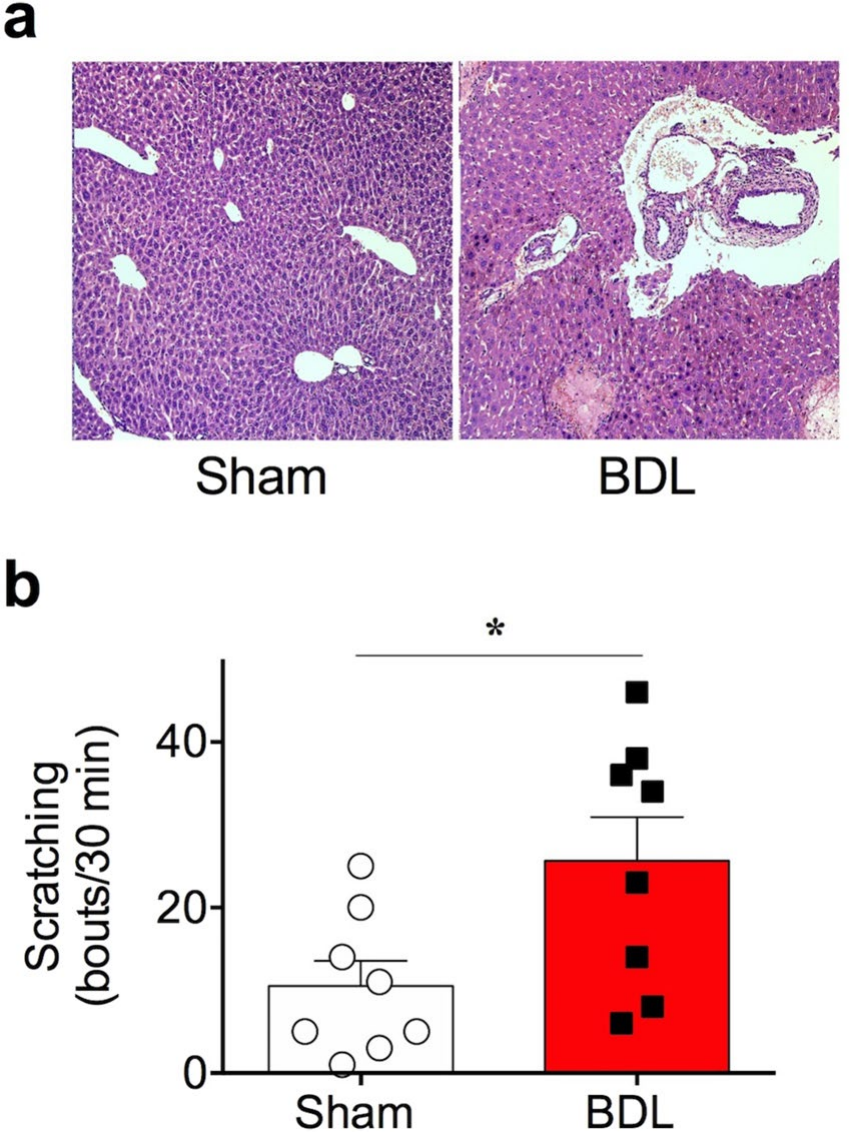
Induction of cholestasis by bile duct ligation. (**a**) Immunohistochemical (H & E) staining of sham and BDL mice liver collected on day 5. Inflammation and cell fibrosis were observed in BDL mice liver. (**b**) Spontaneous scratching behaviour of BDL mice was enhanced on day 5, observed in a 30 min recording (n = 8 in each group) *p < 0.05 (Student’s *t*-test). Results are presented as means ± SEM.

To examine itch under cholestasis conditions, we used an obstructive cholestasis model known as BDL for inducing cholestasis in mice. To verify whether cholestasis was successfully introduced, various parameters that reflect cholestasis were tested. As expected, typical signs of cholestasis such as jaundice, dark urine, and steatorrhea were observed starting from the day after the BDL operation (day 1). Furthermore, BDL mice showed enlargement of gall bladders due to improper bile flow, along with yellow-coloured skin. Histological observation by H & E staining also revealed inflammatory lesions and fibrosis in the liver, which are inevitably associated with most liver diseases such as cholestasis (Fig. 1a). In addition, serum levels of total bile acid, bilirubin, AST, and ALT in BDL mice were significantly higher than those in the sham-operated mice, suggesting that the BDL surgery injured the liver (Table 1). Most importantly, as shown in Fig. 1b, there was a significant increase in spontaneous scratching counts in BDL mice (25.63 ± 5.318 bouts, n = 8, day 5) compared to those in sham-operated mice (10.50 ± 3.047 bouts, n = 8, day 5). Taken together, bile duct obstruction effectively induced cholestasis, which increased spontaneous scratching behaviour in BDL mice.

**Table 1:** Serum levels of liver function markers of bile duct-ligated (BDL) mice (Day 5).

Name	Sham (n = 5)	BDL (n = 7)
Bile acid (μmol/l)	7.400 ± 1.181	>150.0 ± 0.000^***†^
Alanine aminotransferase (ALT; U/l)	28.20 ± 2.672	494.4 ± 85.28^**^
Aspartate aminotransferase (AST; U/l)	97.80 ± 9.583	595.6 ± 58.09^***^
Total bilirubin (mg/dl)	<0.1 ± 0.0^†^	10.50 ± 1.028^***^

**p < 0.01 ***p < 0.001, ^†^Approximate values were used for statistical comparison.

### Transcription of proenkephalin and other related genes were increased in the skin of BDL mice

**Figure 2:**
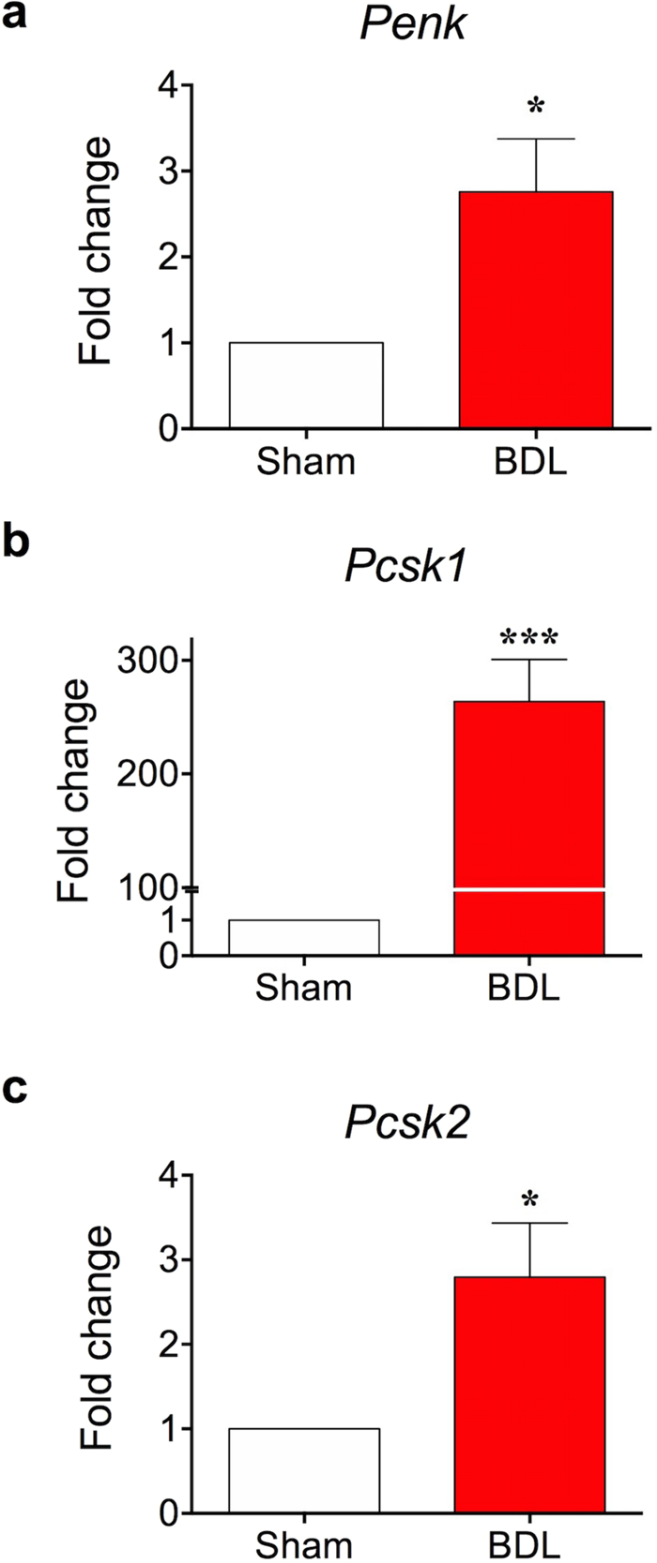
Proenkephalin mRNA expression is elevated in skin of BDL mouse. On day 5, skin tissues were collected from sham and BDL mice and RT-qPCR was performed. Transcriptional levels of *Penk* (**a**), *Pcsk1* (**b**), and *Pcsk2* (**c**) were increased in the skin tissue samples of BDL mice. *p < 0.05, ***p < 0.001 (Student’s *t*-test). Results are presented as means ± SEM.

As mentioned in the Introduction, we postulated that an endogenous peptide, BAM8-22, might be involved in cholestatic pruritus. As BAM8-22 is a cleavage product of its precursor prohormone called proenkephalin, we first investigated changes in proenkephalin (*Penk*) mRNA levels in skins of BDL mice. As shown in Fig. 2a, the *Penk* mRNA level was significantly increased in the skin of BDL mice compared to that of sham-operated mice (2.758 ± 0.6166-fold increase), implying that proenkephalin production was increased in the skin of BDL mice.

Proenkephalin is a prohormone that can be cleaved into various smaller peptides. BAM8-22 is derived from one of the proenkephalin-derived peptides called BAM22P. However, we speculated that high *Penk* transcription may not guarantee enhanced action of BAM8-22, because the latter requires additional proteolytic processing of BAM22P. Hence, we focused on certain proteases, and observed that PCSK (proprotein convertase subtilisin/kexin) was essential for converting BAM22P to BAM8-22. Previous reports show that prohormone convertase is involved in the production of active peptides from proenkephalin^17–19^. Therefore, we also assessed transcriptional changes in two different PCSKs, *Pcsk1* and *Pcsk2*, in the skins of BDL mice. As shown in Fig. 2b,c, the mRNA levels of both *Pcsk1* and *Pcsk2* were significantly higher in BDL mice than in sham-operated mice (*Pcsk1*: 263.7 ± 37.21-fold increase, *Pcsk2*: 2.797 ± 0.6370-fold increase). Therefore, we concluded that *Penk* and *Pcsk1/2* transcription levels were increased in the skins of BDL mice, which may enhance BAM8-22 production.

### *Mrgprx1* expression was increased in the sensory neurons of BDL mice

**Figure 3:**
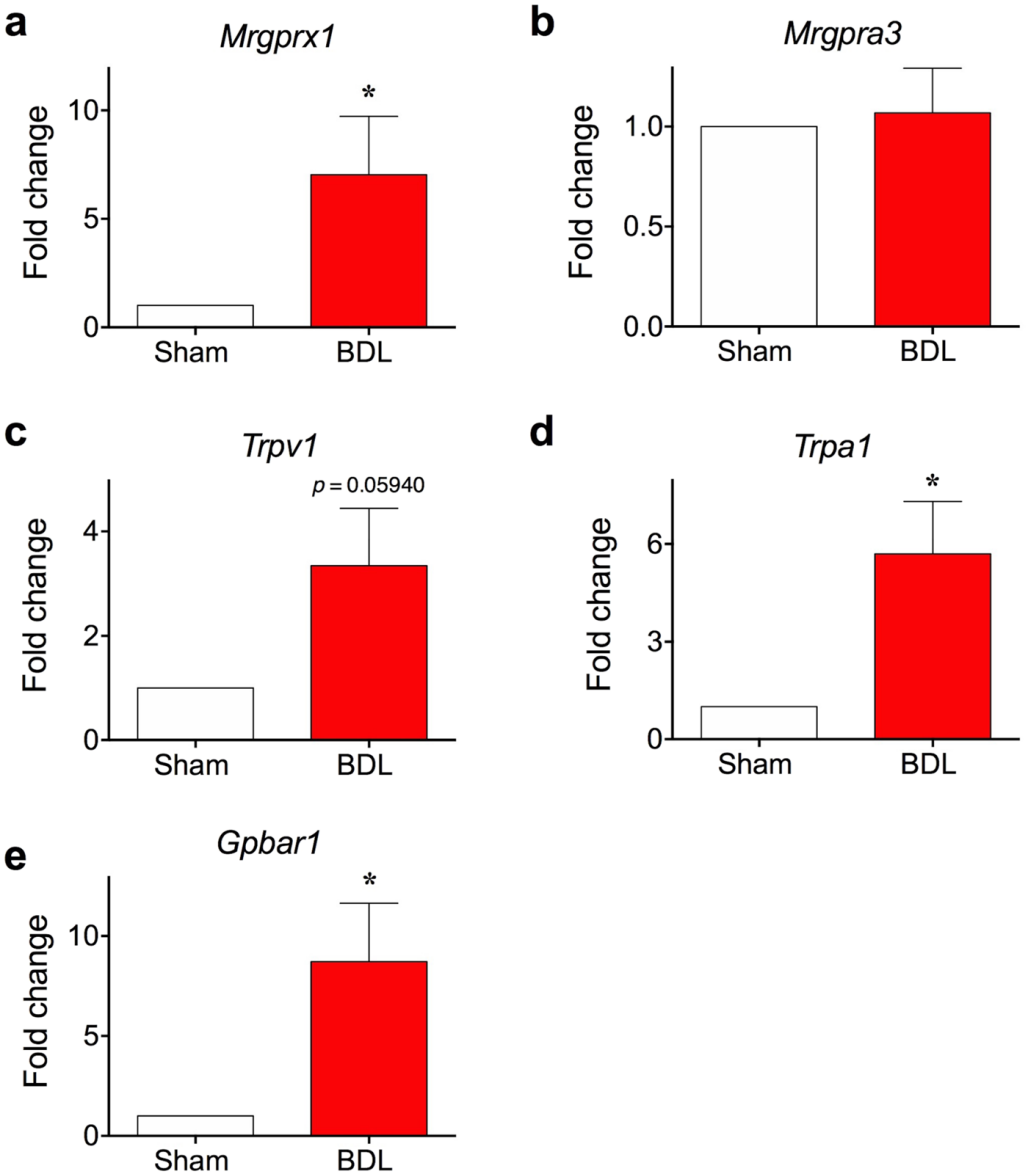
Levels of *Mrgprx1*, *Trpa1*, and *Gpbar1* mRNAs, but not those of *Mrgpra3* and *Trpv1* mRNAs, are elevated in sensory neurons of BDL mice. DRG neurons were also collected on day 5 after surgery and RT-qPCR was performed to determine changes in transcriptional levels of (**a**) *Mrgprx1*, (**b**) *Mrgpra3*, (**c**) *Trpv1*, (**d**) *Trpa1* and (**e**) *Tgr5*. Among these, the expression levels of *Mrgprx1*, *Trpa1*, and *Tgr5* were significantly elevated in DRG neurons of BDL mice. *p < 0.05 (Student’s *t*-test). Results are presented as means ± SEM.

Next, we determined whether there were any changes in the expression of itch-related genes in the sensory neurons of BDL mice. As BAM8-22 is known to specifically activate MRGPRX1^12,20,21^, its mRNA level in DRG of either sham-operated and BDL mice were compared. As shown in Fig. 3a, the mRNA level of *Mrgprx1* in BDL mice was significantly higher than that of sham-operated mice (7.031 ± 2.699-fold increase). In contrast, the transcription level of another itch-related receptor, *MrgprA3*, was not altered in BDL mice (Fig. 3b). Thus, BDL operation specifically increases *Mrgprx1* mRNA level in sensory neurons.

We further investigated alterations in the mRNA levels of certain itch-related ion channels such as *Trpv1* and *Trpa1*. Although *Trpv1* transcription seemed slightly higher in BDL mice than in sham-operated mice, the difference was not significant (*p* = 0.05940, Fig. 3c). On the contrary, the mRNA level of *Trpa1* was significantly higher in the DRG of BDL mice (Fig. 3d).

Finally, we also examined changes in transcription of *Gpbar1* (also known as *Tgr5*), which encodes a receptor for bile acids, because a previous report showed that BDL increases the transcription of this gene^22^. As expected, the mRNA level of *Gpbar1* was significantly increased in DRG of BDL mice (Fig. 3e). Taken these together, we concluded that mRNA levels of *Mrgprx1* and certain other related genes were increased in DRG of BDL mice, which is indicative of enhanced response to BAM8-22 in BDL mice.

### Sensory neurons of BDL mice showed increased BAM8-22-induced intracellular calcium level

**Figure 4:**
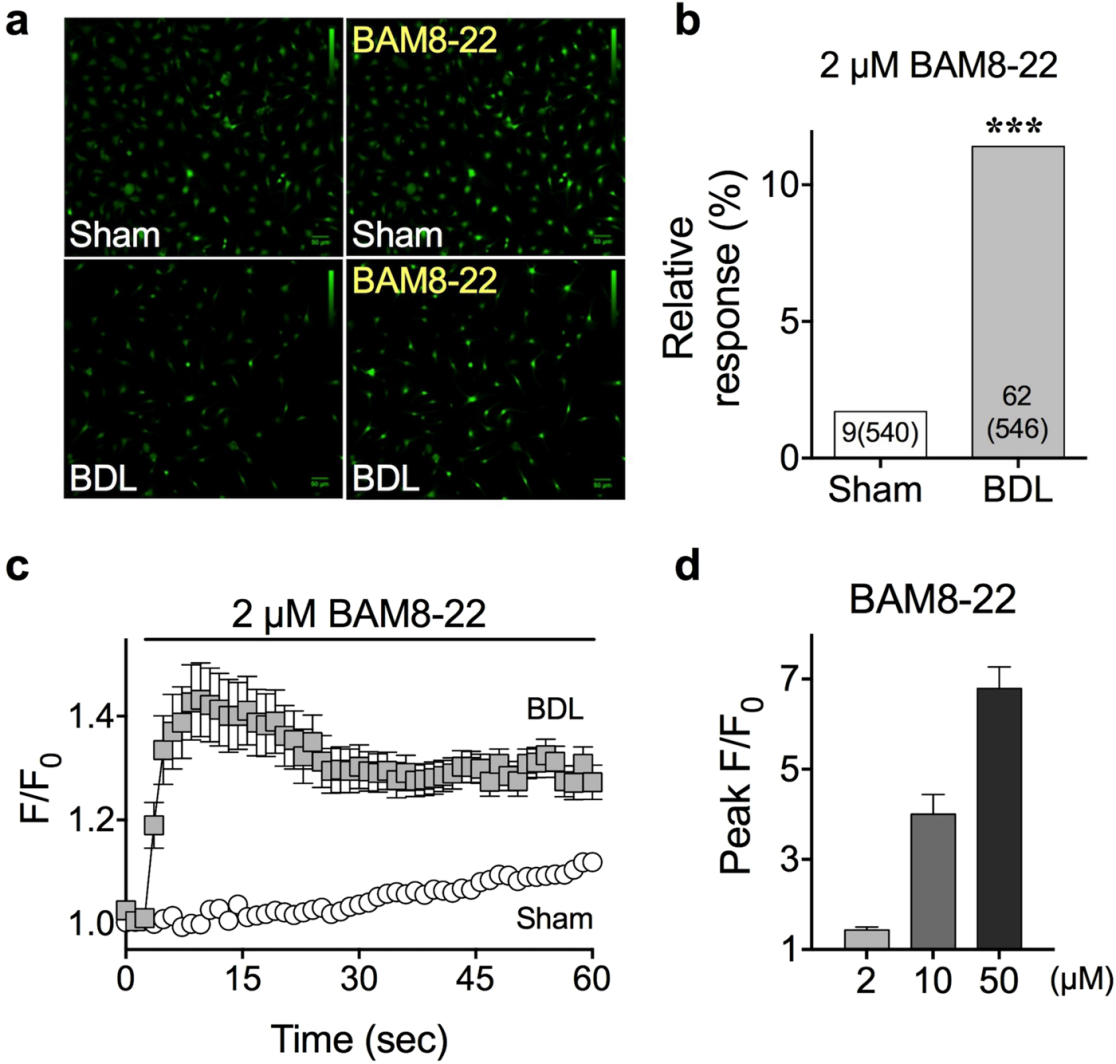
BAM8-22-induced intracellular calcium level was increased in sensory neurons of BDL mice. On day 5, DRG neurons were cultured and treated with 2 μM BAM8-22. (**a**) Representative calcium imaging results before and after BAM8-22 treatment on cultured DRG neurons of either sham or BDL mice. (**b**) Bar graph shows the relative response (%) and number of responsive cells compared to total cells after 2 µM BAM8-22 treatment. The response of BDL DRG neurons was higher than those from the sham. (**c**) A time-course graph of calcium imaging after treatment of DRG neurons of both sham and BDL mice with 2 µM BAM8-22. (**d**) Comparisons of the peak of intracellular calcium level (peak F/F_0_) after treatment of BDL DRG neurons with different concentration of BAM8-22. ***p < 0.001 (Fischer’s exact test). Results are presented as means ± SEM.

We examined functional changes related to BAM8-22 in the sensory neurons of BDL mice. Towards this objective, primary cultures of DRG from either sham or BDL mice (day 5) were prepared, and responses to BAM8-22 treatment were determined by calcium imaging. When 2 μM BAM8-22 was applied on the DRG neurons, the intracellular calcium level was significantly increased in the DRG of BDL mice than in those of sham-operated mice (Fig. 4a,c). Moreover, DRG neurons of BDL mice not only showed increased intracellular calcium level, but also a dramatic increase in the number of BAM8-22-responsive neurons (Fig. 4b, sham: 1.7% [9 out of 540 cells], vs. BDL: 11.4% [62 out of 546 cells]). When a time course of BAM8-22-mediated intracellular calcium level was analysed (Fig. 4c), we observed that the peak ratios (F/F_0_) increased in a dose-dependent manner (Fig. 4d). Thus, these results strongly imply that MRGPRX1 expression increases in BDL mice, which might increase the response to BAM8-22 treatment.

### Scratching behaviour of BDL mice was potentiated after BAM8-22 administration

**Figure 5:**
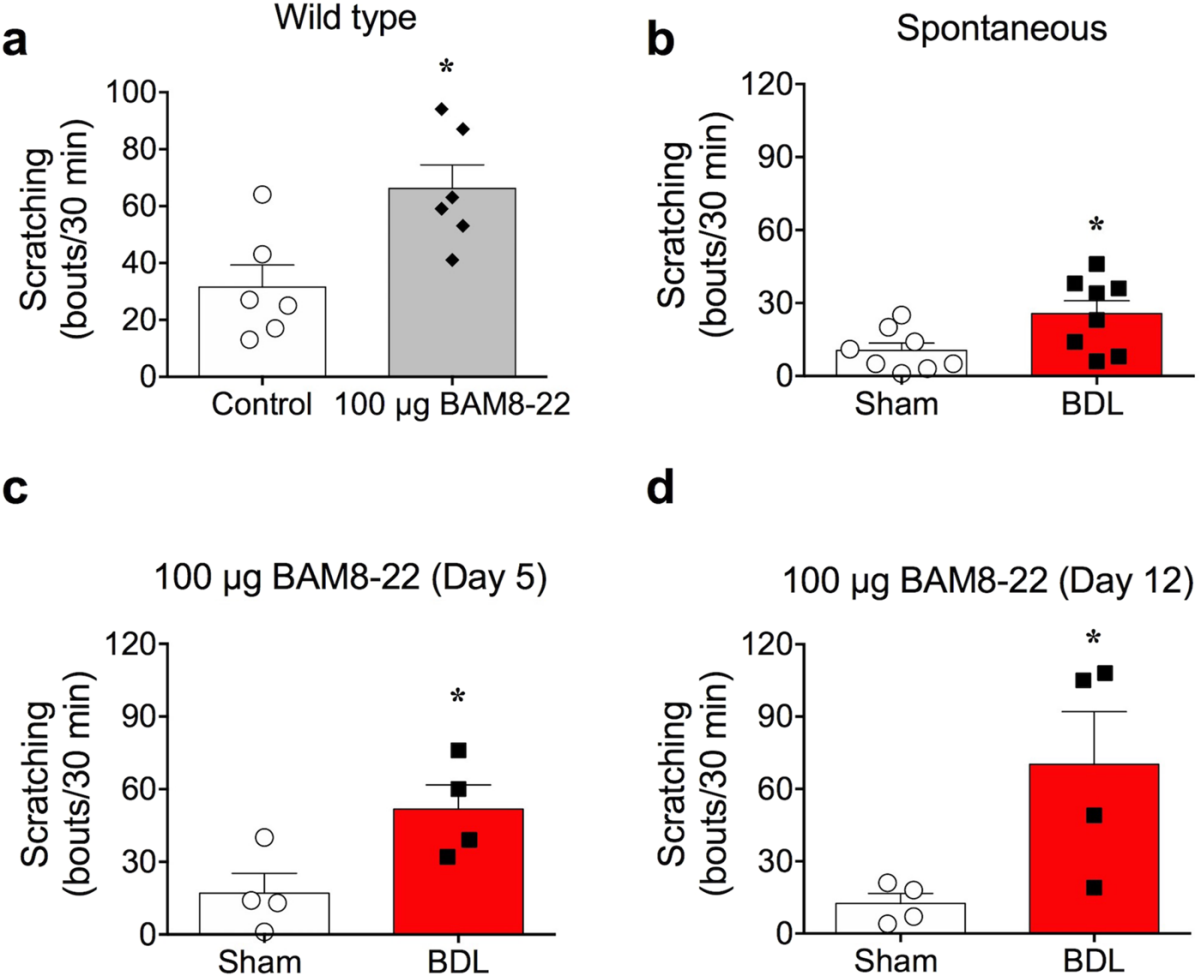
Synergistic effect of BAM8-22 on scratching behaviour in BDL mice. (**a**) One hundred micrograms BAM8-22 was injected on mice cheeks, which induced scratching behaviour in wild type mice, confirming that BAM8-22 acts as a pruritogen (n = 6, each group). (**b**) Increased spontaneous scratching behaviour in BDL mice on day 5 after surgery showed that scratching is one of the major symptoms of cholestasis (n = 8 in each group). A synergistic effect was observed when 100 µg BAM8-22 was injected into the cheek of BDL mice compared to that in sham mice on day 5 (**c**) and day 12 (**d**), respectively (n = 4, each group). *p < 0.05 (Student’s *t*-test). Results are presented as means ± SEM.

If the increase in MRGPRX1 expression in the sensory neurons of BDL mice is authentic, BDL mice might react more sensitively to its agonist, BAM8-22. While it has been reported that BAM8-22 administration induces scratching behaviour^12,20,21^, we verified whether BAM8-22 indeed works as a pruritogen when administered to wild type mice. As shown in Fig. 5a, injection of 100 µg BAM8-22 under the cheek successfully induced scratching behaviour, whereas PBS injection did not (BAM8-22: 66.17 ± 8.320 bouts, n = 6 *vs*. control: 31.50 ± 7.753 bouts, n = 6). Therefore, we confirmed that BAM8-22 is indeed a pruritogen that can induce scratching behaviour in mice.

Previously, we showed that BDL mice show increased spontaneous behaviour compared to sham-operated mice (Fig. 5b, identical data to Fig. 1b). Nonetheless, we further examined whether BDL mice are more sensitive to BAM8-22, as increased expression of MRGPRX1 in the sensory neurons are anticipated in BDL mice. Therefore, 100 µg BAM8-22 was injected under the cheek of BDL mice, and scratching behaviour was investigated. As shown in Fig. 5c, BAM8-22 strongly increased scratching bouts in BDL mice on day 5. Furthermore, the total number of scratching bouts was higher in day 12 BDL mice than in sham-operated mice. This suggests that MRGPRX1 expression was indeed increased in sensory neurons of BDL mice, as was evident from the increased scratching behaviour after BAM8-22 injection. In conclusion, BDL mice possessed more active MRGPRX1 in sensory neurons, which might cause increased sensitivity to BAM8-22. In addition, it is worth mentioning that BAM8-22 injection did not evoke noticeable increase of scratching bouts in “sham” mice (undergone the same surgery of BDL except bile duct ligation, Fig. 5b–d) when compared to “wild type” mice (no surgery, Fig. 5a). It is speculated that the surgery in sham group may cause post-operative pain which can inhibit spontaneous and BAM8-22-induced itch in Fig. 5b–d.

### BAM8-22 might be increased in the skin of BDL mice

As BAM8-22 specifically activates and induces intracellular calcium increase through stimulation of MRGPRX1, we applied skin homogenates of either BDL or sham-operated mice onto *Mrgprx1*-transfected HeLa cells and compared their responses using calcium imaging.

**Figure 6:**
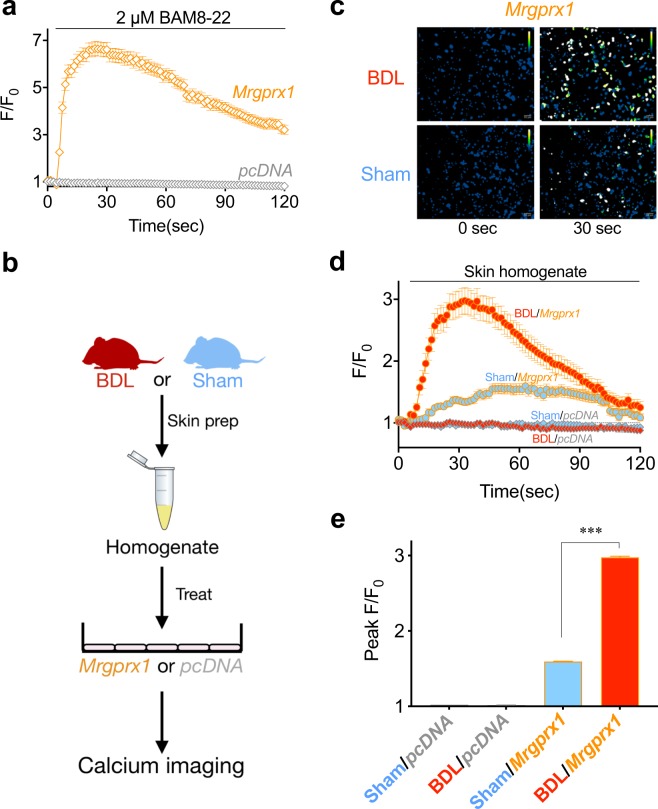
BDL skin homogenate increased intracellular calcium through *Mrgprx1*. (**a**) Application of 2 µM BAM8-22 induced significant intracellular calcium increase, which implies that the transient expression of *Mrgprx1* in HeLa cells was functional. (**b**) A schematic illustration of the experiment carried out for calcium imaging with skin homogenates. (**c**) Representative pseudo-coloured ratiometric images of sham or BDL skin homogenate treatment on HeLa cells expressing *Mrgprx1* or *pcDNA3*.*1* (*“pcDNA”*). (**d**) Time-course graphs of calcium imaging after treatment of skin homogenates collected from either sham or BDL mice on *Mrgprx1- or pcDNA-*transfected HeLa cells. The BDL skin homogenate significantly increased intracellular calcium level compared to that of sham. (**e**) Summary bar graphs showing the peak responses from either sham or BDL skin homogenate treatment onto *Mrgprx1-* or pcDNA-transfected HeLa cells. ***p < 0.001 (one-way ANOVA, Dunnett’s multiple comparison test). Results are presented as means ± SEM.

First, we checked whether mouse *Mrgprx1* inserted into the pcDNA3.1 vector is suitable for detection of BAM8-22 using calcium imaging. As shown in Fig. 6a, when *Mrgprx1* was transiently transfected in HeLa cells (“*Mrgprx1*/HeLa”), application of 2 µM BAM8-22 induced significant increase in intracellular calcium level, indicating that MRGPRX1 was successfully and transiently expressed in HeLa cells. On the contrary, cells transfected with the pcDNA3.1 vector did not show noticeable intracellular calcium increase even after 2 µM BAM8-22 treatment (Fig. 6a). Thus, it was verified that BAM8-22 can specifically induce intracellular calcium in *Mrgprx1*/HeLa.

Next, skin homogenates collected from either BDL or sham-operated mice were applied on *Mrgprx1*/HeLa cells. As shown in Fig. 6c–e, treatment with BDL skin homogenate caused robust increase in intracellular calcium in *Mrgprx1*/HeLa cells, which was significantly higher than that of sham skin homogenate.

Finally, we performed immunofluorescence assay with BAM22P antibody in the skin of either sham or BDL mice. It should be mentioned that there was no commercially available antibody of BAM8-22, thus we had to detect presence of BAM22P, a direct precursor of BAM8-22. As a result, it was found that there were marked increase of fluorescence intensity in the epidermal region from BDL mice than that of sham (Fig. S1), suggesting that there is a high chance of increased BAM8-22 production in BDL condition.

Taken together, these results strongly suggest that BDL mice may preferentially produce more BAM8-22 than sham-operated mice.

### CDCA could be a major bile acid that increases PENK expression in human keratinocytes

As all the aforementioned results were obtained from BDL mice, we sought to investigate whether bile acids can also upregulate *PENK* in human skin. Generally, bile acids are a mixture of molecules synthesized in the liver as unconjugated or conjugated forms. Therefore, we speculated that not all bile acids have the same effect on *PENK* regulation. To investigate this, the effect of various individual bile acids on *PENK* regulation was verified in HaCaT cell line, which is derived from human keratinocytes. Specifically, HaCaT cells were treated with various primary and secondary bile acids, and changes in *PENK* transcription after 24 h of treatment were determined.

**Figure 7:**
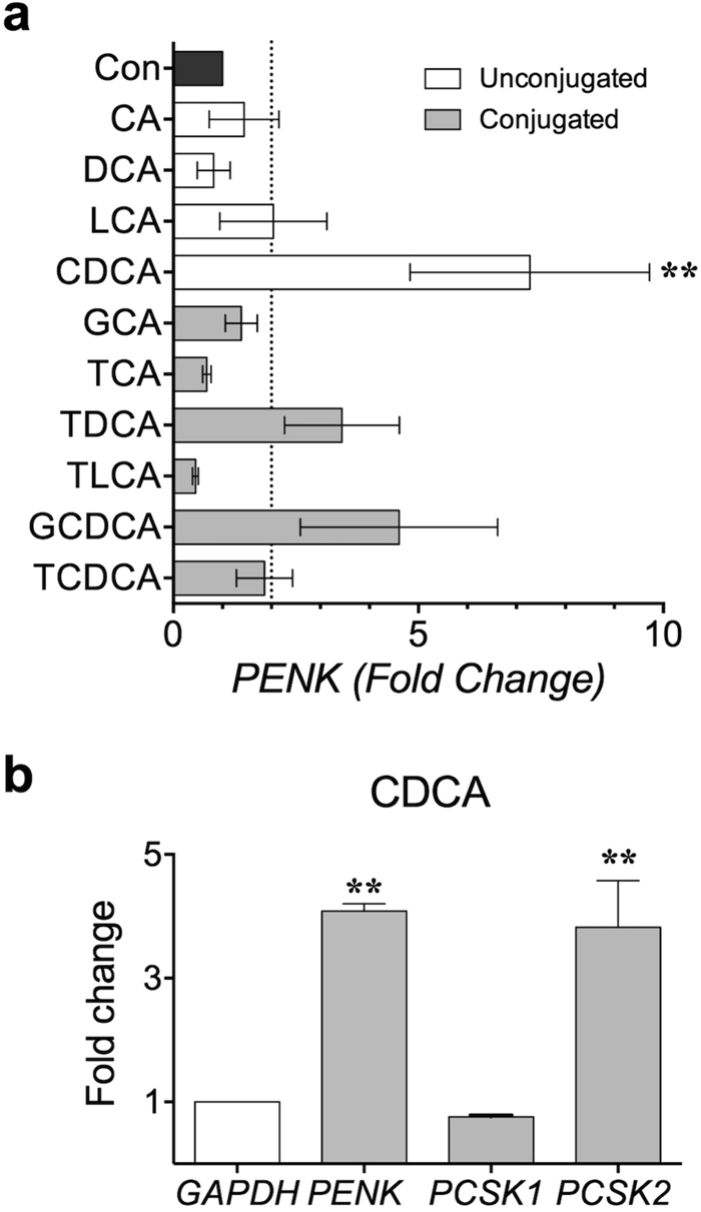
CDCA increased the mRNA level of proenkephalin (*PENK*) and the related enzyme *PCSK2*, but not that of *PCSK1* in HaCaT cells. (**a**) Effect of conjugated and unconjugated bile acids on *PENK* regulation when HaCaT cells were treated for 24 h. Only CDCA significantly increased the level of proenkephalin, **p < 0.01 (one-way ANOVA, Dunnett’s multiple comparison test). (**b**) Effect of CDCA on *PENK*, *PCSK1*, and *PCSK2*. Results show only significant increase in *PENK* and *PCSK2* expression. **p < 0.01 (one-way ANOVA, Tukey’s multiple comparison test). Results are presented as means ± SEM.

Among 11 different bile acids, we observed that only CDCA significantly increased *PENK* expression in HaCaT cells (Fig. 7a). Surprisingly, none of the other primary and secondary bile acids induced significant changes in *PENK* transcription. Therefore, CDCA could be a major bile acid that increases *PENK* level. Furthermore, the transcriptional changes in *PCSK1* and *PCSK2* were also verified. We observed that CDCA treatment increased only *PCSK2* expression significantly in HaCaT cell line (Fig. 7b). Therefore, these results imply that CDCA possibly upregulates *PENK*, which in turn increases production of BAM8-22 in human keratinocytes. In other words, these results distantly suggest that BAM8-22 may be a possible pruritogen of human cholestasis.

## Discussion

Itch has been defined as an “unpleasant skin sensation that elicits the desire or reflex to scratch”^23^. Generally, dermatological diseases such as atopic dermatitis accompanies chronic itch. However, non-dermatological conditions such as infection, nervous system disorders^13^, systemic disorders, haematological diseases^24,25^, and chronic liver diseases such as cholestasis, may also present pruritus. Antihistamines are often clinically prescribed to cure or alleviate pruritus. However, owing to the existence of histamine-independent itch pathways, antihistamines are mostly ineffective for various chronic itch conditions such as cholestasis^2,26,27^. Till date, various histamine-independent pruritogens have been reported, such as chloroquine^28^, β-alanine^29^, TSLP^30^, IL-31^31^, SLIGRL-NH_2_^32^, and BAM8-22^32^.

While BAM8-22 is a well-known pruritogen^12,13,20,21^, its involvement in cholestasis has never been reported. We observed high *Mrgprx1* mRNA level, which encodes a specific receptor for BAM8-22, in the DRG neurons of BDL mice (Fig. 3a) for the first time. In addition, calcium imaging showed increased function of MRGPRX1 in DRG neurons of BDL mice (Fig. 4a–c). Furthermore, the effect of elevated MRGPRX1 function in BDL mice might be reflected in the synergistic scratching behaviour after BAM8-22 treatment (Fig. 5c,d). More importantly, treatment with skin homogenate of BDL mice increased intracellular calcium level in *Mrgprx1*/HeLa cells, strongly suggesting that BAM8-22 could be a pruritogen responsible for cholestasis.

We also observed increase in *Trpa1* levels in the DRG neurons of BDL mice, which is in agreement with the previous report showing that TRPA1 is required for itch induced by BAM8-22^13^. Meanwhile, *Trpv1* level was not increased in BDL mice when analysed by student’s *t*-test (Fig. 3c). Although this data is in agreement with the finding that *Trpv1* is not required for BAM8-22-induced neuronal excitation^13^, there is a possibility that the increase of *Trpv1* could be a false negative, especially when the *p* value was considered (*p* = 0.05945). Thus, chances are that TRPV1 may still play an adjunctive role in BAM8-22-induced itch. In fact, there is an earlier report that potentiation of the TRPV1 contributes to pruritogenesis in a rat model of liver disease^33^. Therefore, a separate further study is mandatory to clarify the controversial role of TRPV1 in cholestatic pruritus.

As the BDL model is not the only method that can induce cholestasis in mice, one may argue whether BDL is an appropriate animal model for investigating cholestatic pruritus. For example, mice with knockouts in *Gpbar1*^−/−^^34^, *Spgp*^−/−^^35^, *Cyp7*^−/−^^36^, and *Fxr*^−/−^^37^ readily exhibit cholestasis. However, we deliberately excluded these knockout mice because we did not want to restrict the cause of cholestasis to specific genes in the present study. Injection of specific drugs is another alternative method for inducing cholestasis. Among various drugs, two compounds, namely α-naphthyl-isothiocyanate (ANIT) and 17α-ethynylestradiol (EE) are most widely used for inducing cholestasis. Although cholestasis induced by either ANIT or EE is feasible and reliable, this method was considered unsuitable for the present study. For EE, this is because that EE-injected mice did not show any enhanced spontaneous scratching behaviour^34^. For ANIT, on the other hand, enhanced spontaneous scratching behaviour was not consistently found, as one report found no increase of scratching behaviour^34^, whereas a recent work claimed enhanced scratching behaviour^38^. As our BDL model consistently exhibited increased spontaneous scratching behaviour (Fig. 1b), it was a critical factor for us to select as a cholestasis model of choice in the present study. Taken together, we believe that our BDL mouse is an appropriate cholestasis model for investigating the underlying mechanism of cholestatic pruritus.

Although the present study suggests that BAM8-22 and MRGPRX1 are involved in the mechanism underlying cholestatic pruritus, other mediators may still be involved. For example, evidence indicates that lysophosphatidic acid (LPA) and autotaxin (the enzyme that produces LPA) are responsible for the pruritus in cholestasis^39,40^. Specifically, serum autotaxin activity was increased in patients with cholestatic pruritus^40^, and injection of LPA caused scratching behaviour in mice^39^. However, the role of LPA in either cholestasis patients or cholestatic mouse model has not yet been confirmed, implying that further studies are required to elucidate the role of LPA and/or autotaxin^4^. Serotonin is another candidate cholestasis-associated pruritogen. Tian and colleagues observed elevated levels of serotonin in the spinal cord and skin of BDL rats^14^, and claimed that peripheral and central serotonin systems participate dynamically in pruritus under cholestasis; therefore, targeting of serotonin receptors may be an effective treatment for cholestatic pruritus. Indeed, many serotonin antagonists such as ondansetron^27,41^, sertraline^42^, and dronabinol^43^ have been used to treat pruritus in cholestasis. However, since serotonin is associated with both itch and pain, explaining cholestatic itch-related pathways solely on the basis of serotonin action would be challenging. Recently, it was reported that bilirubin could also play as a pruritogen in the ANIT-induced animal model^38^. It was further found that human *MRGPRX4* (or mouse *MrgprA1*) could be a molecular entity responsible for bilirubin-induced itch sensation. Overall, it appears that understanding cholestatic pruritus would be entirely challenging task due to existence of various multiple pruritogens responsible for cholestatic pruritus.

In this regard, the current study presents a novel cholestatic itch pathway from distinct perspectives. The conclusion, that BAM8-22, a proenkephalin-derived pruritogen, mediates cholestatic pruritus, stemmed from various previous reports. For instance, studies showed that hepatic concentration of opioids derived from proenkephalin increased in a rat model of cholestasis^44^. In addition, the adrenal secretion of BAM22P, a direct precursor of BAM8-22, was also increased in rats with acute cholestasis^45^. Furthermore, clinical and experimental observations from cholestatic liver disease revealed elevated concentration of plasma enkephalin, which is also derived from proenkephalin^46^. Importantly, proenkephalin mRNA level in the skin was increased both in patients with obstructive jaundice and rat models of cholestasis^47^. These results indicated a novel pathway via which BAM8-22 mediates cholestatic pruritus in BDL mice.

It is noteworthy that reports claiming the increased production of proenkephalin (PENK) in “human” cholestatic livers are lacking. However, it should also be mentioned that the expression of methionine enkephalin (Met-enkephalin), another PENK derivative, was strongly detected in the liver of patients with primary biliary cirrhosis^48^. This implies that the adult human cholestatic liver may produce high levels of PENK. Furthermore, (a) the unequivocal detection of *Penk* in our BDL mice liver, (b) the presence of Met-enkephalin immunoreactivity in cholestatic rat^46^ and human liver^48^, and (c) the accumulation of PENK-derived endogenous opioids in livers of rats with cholestasis indicate the importance of PENK in human cholestasis^44^. Several reports indicate that there may be a possible link between opioid receptors and MRGPRX1^49^. This is especially true according to a previous report which claimed MrgC11 (another name for MRGPRX1) and µ-opioid receptor are colocalized in sensory neurons to induce morphine analgesia^50^. Thus, we investigated a possibility whether the action of BAM8-22 can be affected by µ-opioids. However, it was found that naltrexone – an antipruritic agent with µ-opioid receptor antagonist activity – did not inhibit BAM8-22-induced intracellular calcium increase in cells expressing MRGPRX1 (Fig. S2). Moreover, neither naltrexone nor enkephalin (a µ-opioid agonist also derived from PENK) had effect on MRGPRX1 (Fig. S2). These data indicate that µ-opioids are not likely to directly affect the function of MRGPRX1. Therefore, it is assumed that the pruritogenic effect of BAM8-22 is barely affected by direct action of µ-opioids on MRGPRX1. However, there are plenty of possibilities that opioid receptors are interrelated to BAM8-22-induced signalling pathway. Therefore, further in-depth study is necessary to reveal the putative interactions between MRGPRX1 and opioid receptors.

Humans differ significantly from mice with respect to bile acids. In humans, CA and CDCA are the major bile acids. In contrast, CA and β-muricholic acid are the key bile acids in mice, and CDCA is not considered a major bile acid in mice. Thus, the cholestasis introduced in BDL mice may not completely recapitulate human cholestasis. However, the difference in bile acid composition might be limited to “healthy” mice because the level of CDCA in serum may rise up to millimolar range (4.5 ± 0.4 µmol/ml = mM) in BDL mice^51^. Therefore, although CDCA is not considered as a major bile acid in normal healthy mice, it is plausible that CDCA could be a major bile acid in BDL mice. Because upregulation of *PENK* was observed after CDCA treatment on human keratinocytes (Fig. 7a), chances are that CDCA indeed has ability to upregulate *PENK* in human skins.

CDCA is strongly associated with liver injury. This is distantly supported by the observation that the liver injury model can be mimicked by CDCA administration to hamsters^52^. However, it is unclear whether CDCA *per se* is able to cause pruritus. Nevertheless, our observations suggest that CDCA might be involved in cholestatic pruritus, probably by increasing production of PENK, and subsequently BAM8-22. Although secondary bile acids such as DCA and LCA may also be involved in cholestatic pruritus, the concentration of secondary bile acids in the plasma and the liver did not dramatically increase in humans with cholestasis or in animal models of cholestasis^53^. This could be due to the fact that secondary bile acids are formed in the intestine from primary bile acids; however, the intestinal concentration of primary bile acids normally decreases under cholestasis because of reduced biliary excretion. Although further studies are required, the current results imply that increase of BAM8-22 production in the skin is a possible scenario under cholestatic condition.

## Conclusion

**Figure 8:**
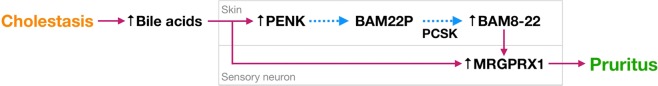
The proposed itch signaling pathway in cholestasis. Cholestasis increases bile acids including CDCA, which accumulate in the skin. This in turn increase the mRNA levels of PENK, which facilitates enhanced production of BAM22P. BAM22P can be further cleaved into the active peptide BAM8-22 via the action of enzymes such as PCSK1 and PCSK2. Furthermore, expression of MRGPRX1, a specific receptor for BAM8-22, also increased in DRG of cholestasis model mouse, resulting in augmented transmission of the cholestatic pruritic signal to sensory neurons of the brain.

In the present study, we observed that BAM8-22, a cleaved product of proenkephalin, plays important roles in cholestasis-associated pruritus, probably because of CDCA. Specifically, both increased action of BAM8-22 in the skin and enhanced sensitivity of MRGPRX1 in the sensory neuron may augment pruritus, which in turn leads to enhanced scratching behaviour in BDL mice (Fig. 8). We believe that the present study may shed a light on novel itch signalling pathways in cholestasis and provide a way to alleviate the intractable itch sensation.

## Materials and Methods

### Reagents

BAM8-22 was purchased from Tocris (Bristol, UK) and dissolved in 1× phosphate buffered saline (PBS). Cholic acid (CA), tauro-cholic acid (TCA), glycol-cholic acid (GCA), chenodeoxycholic acid (CDCA), tauro-chenodeoxycholic acid (TCDCA), glycol-chenodeoxycholic acid (GCDCA), deoxycholic acid (DCA), tauro-deoxycholic acid (TDCA), lithocholic acid (LCA), and tauro-lithocholic acid (TLCA) were purchased from Sigma Aldrich (St. Louis, Missouri, USA). Protease inhibitor cocktail (PIC) was purchased from Bio Vision (Milpitas, CA 95035 USA).

### Animals

All experimental animal protocols were approved by the institutional animal care and use committee of the Gachon University (GIACUC-R2017035) and were performed in accordance with the guidelines for the Care and Use of Laboratory Animals. Mice were regularly observed after BDL surgery with care. Most importantly, distensions of the abdomen or ascites formation after the surgery were set as humane endpoints for euthanasia.

### Bile duct ligation (BDL) surgery

Nine-week-old male ICR mice were purchased from Koatech (Pyeongtaek, Gyeonggi-do, Korea) and surgery was performed after one-week acclimatization. The mice were anesthetized with a mixture of Zoletil-Rompun (Vibac S.A, France and Bayer Gyeonggi-do, Korea respectively). Next, the abdominal area of the mouse was shaved and sterilized with 70% ethanol. The abdomen was opened in the midline with an approximately 2-cm long laparotomy after cutting the cutis plus fascia. Then, dissection of the connective tissue on top of the peritoneum was performed using the scissors as spreader. The peritoneum was cut along the linea alba to open the peritoneal cavity, which was enlarged by inserting a holding suture in the sternum, raising the filament of the suture, and fixing. Later, the operation area was spread by inserting a retractor in the peritoneal cavity. The liver was lifted with a moisturized saline cotton swab such that its ventral side adhered to the diaphragm and the hilum was clearly visible, exposing the bile duct by caudal movement of the gut. Then, the bile duct was separated from the flanking portal vein and hepatic artery using micro-serrated forceps, placing the 5–0 suture around the bile duct and securing it with one surgical knot (single-knot ligation method). While tying the knots, the tractive force was increased continuously to ensure effective obstruction without severing the bile duct. The ends of the sutures were cut, the sternum was lowered, and the retractor was removed. The peritoneal cavity was rinsed with saline and the abdominal organs were replaced in their anatomical positions. Finally, the abdominal layers (peritoneum and cutis plus fascia) were closed with separate running sutures of 5–0 mersilk. The ends of the sutures were cut, and the operation area was sterilized with a gauze swab moistened with ethanol solution. The mice were allowed to recover in a cage warmed by an infrared lamp until they were fully awake and active. Afterwards, the mice were moved to a normal cage and provided *ad libitum* access to water and food. The animals were monitored at regular intervals. Mice with ascites and other complications were excluded from the study.

### Measurement of serum biochemical markers

Blood samples were collected for measurement of serum biochemical markers such as bile acids, aspartate aminotransferase (AST), alanine aminotransferase (ALT), and total bilirubin. Blood was collected from the sham-operated and BDL mice and incubated undisturbed at room temperature for an hour. Then, the serum was separated by centrifugation at 4 °C, 13000 RPM for 10 min. Samples were sent to GreenCross LabCell (GCLC, Yongin, South Korea), for determination of the aforementioned serum biochemical markers.

### Histopathological examination

The liver was retrieved for histochemical tests on day 5. The liver portions of the sham-operated and bile duct-ligated mice were fixed in the 10% formalin. Then, the liver sections were stained with haematoxylin and eosin (H & E staining) and examined microscopically. Histological findings were assessed by observing the shape and structure of the liver sections.

### Reverse transcription-quantitative polymerase chain reaction (RT-qPCR)

Total RNA was extracted from the dorsal root ganglia (DRG) and skin tissues using Trizol (Invitrogen, Carlsbad, California, USA) according to the manufacturer’s instructions. Total RNA (1 µg) was reverse-transcribed using PrimeScript RT reagent (Takara Bio Inc., Kusatsu, Shiga, Japan). RT-qPCR was performed using SYBR Green (Takara). Primers used are listed in Tables S1 and S2. Each target gene expression level was normalized to that of *GAPDH*.

### Primary culture of DRG

DRG neurons were primarily cultured as described previously^54^. Ganglia were incubated for 30 min at 37 °C with 1 mg/ml collagenase (Worthington Biochemical, Lakehold, NJ, USA), followed by additional 30 min incubation at 37 °C with 2.5 mg/ml trypsin (Gibco, Gangnam, Korea). Then, they were cultured in neurobasal medium (NBM), containing 10% fetal bovine serum, 50–100 ng/ml nerve growth factor (Invitrogen, Gaithersburg, MD), and 100 U/ml Zell Shield (Minerva Biolabs, Berlin, Germany), plated on poly-L-lysine-treated 8-well glass slides, and incubated for 4 or more days in the presence of 95% humidity and 5% CO_2_ at 37 °C_._

### Cell culture and transfection

HeLa cells were cultured in Dulbecco’s modified Eagle’s medium (DMEM) containing 10% fetal bovine serum (FBS) and 1% Zell Shield (Minerva Biolabs, Berlin, Germany). Grown cells were transfected using the Viafect transfection reagent (Promega, Madison, WI, USA) according to the manufacturer’s instructions. Calcium imaging experiments were performed 24 h after the transfection. Similarly, human keratinocyte (HaCaT) cell lines were cultured in DMEM containing 10% heat inactivated FBS and 1% penicillin/streptomycin (Welgene, Gyeongsangbuk-do, Korea).

### Skin homogenate preparation

Sham and BDL mice on day 5 were anesthetized to obtain 50 μg of skin samples from the abdominal region. The samples were collected in tubes containing 1 ml of ice-cold PBS solution and 50 μl of protease inhibitor complex. The samples were then homogenized for 7–10 min, and centrifuged at 4 °C, 14,000 rpm for 20 min to remove debris. The final supernatant homogenate was dissolved in NBS solution (10%) for further calcium imaging experiments.

### Calcium imaging

Intracellular calcium was detected using a calcium-specific dye and a fluorescence microscope (ECLIPSE Ti-U; Nikon, Tokyo, Japan). Briefly, cells were loaded with 5 μM Fluo-3/AM (Invitrogen, Carlsbad, CA, USA) and incubated for 40 min at 37 °C. Then, the cells were washed with 1× NBS solution (140 mM NaCl, 5 mM KCl, 2 mM CaCl_2_, 0.5 mM MgCl_2_, 10 mM glucose, 5.5 mM HEPES, adjusted to pH 7.4). The excitation wavelength was 488 nm, and emitted fluorescence was measured at 515 nm. The microscopic images were recorded for 2 min at an interval of 1.5 s using the connected computer. Changes in intracellular calcium levels were expressed as F/F_0_ ratios, where F indicates fluorescence intensity at a given time and F_0_ is the fluorescence intensity at 0 s. Images were analysed using ImageJ (NIH, 1.46r) with custom scripts for automatic cell counts and ratiometric image production.

### Mouse scratching behaviour test

Nine-week-old ICR male mice were purchased from Koatech (Pyeongtaek, Gyeonggi-do, Korea). One hundred micrograms BAM8-22 was injected in the right cheek of mice, and scratching behaviour was video-recorded, whereas scratching bouts were counted later. Scratching was counted as one bout when mice scratched using their hind limbs, with their soles placed on the floor.

### Statistical analysis

All the values in the present study were expressed as mean ± standard error of mean (SEM). One-way analysis of variance (ANOVA) or unpaired Student’s t-test was used for statistical analysis, and *p* values < 0.05 indicated statistical significance. Fisher’s exact test was used to compare the responsiveness of the primarily cultured DRG neurons.

## Supplementary information

Supplementary Information

## Data Availability

The datasets analyzed during the current study are available from the corresponding author on reasonable request.
