# Supplementary material for: BAM8-22 and its receptor MRGPRX1 may attribute to cholestatic pruritus
Source: Sci Rep. 2019 Jul 26;9:10888. doi: 10.1038/s41598-019-47267-5 (PMC6659683; doi:10.1038/s41598-019-47267-5)
Supplement: Supplementary file 1 — Supplementary Information [file 41598_2019_47267_MOESM1_ESM.pdf]

Supplementary Information

BAM8-22 and its receptor MRGPRX1 may attribute to cholestatic pruritus

Babina Sanjel[1,2], Han-Joo Maeng[1,2], Won-Sik Shim[1,2 *]

[1]College of Pharmacy, Gachon University, Hambakmoero 191, Yeonsu-gu, Incheon 21936, Republic of Korea

[2]Gachon Institute of Pharmaceutical Sciences, Hambakmoero 191, Yeonsu-gu, Incheon 21936, Republic of Korea

Immunofluorescence analysis

On day 5, skin samples from sham and BDL mice were obtained and fixed in 10% formalin. The tissue samples were then prepared for paraffin embedding and cut into sections and slide mounted. The slides with the tissue samples were dewaxed and rehydrated with Xylene, serially diluted ethanol and with distilled water at last. The tissue sections were washed with PBS and used 4% paraformaldehyde for 10 minutes fixation. 1% Hydrogen peroxide was used to suppress endogenous peroxidase activity whereas 1% FBS on 0.3% Triton X-100 was used for blocking. Skin tissue sections were then incubated with primary antibody, Anti-BAM22P antibody (Abcam, Cambridge, UK) 1:50 dilutions were used for overnight incubation at 4°c. After washing the tissue samples with PBS, they were treated with secondary antibody, Goat Anti-Rabbit IgG-H&L Alexa Fluor®488 (Abcam, Cambridge, UK) 1:1000 dilutions were used for 2 hours incubation at room temperature. Then the samples were washed with PBS and finally VECTASHIELD (Vector laboratories, Burlingame, CA, USA) mounting medium was applied and protected with the cover slips. The slides were visualized, and fluorescent images were obtained using Leica DMi8 inverted microscope (Leica Microsystem Ltd., Wetzlar, Germany).

Cell culture and transfection

HEK293T cells were cultured in Dulbecco's modified Eagle's medium (DMEM) containing 10% fetal bovine serum (FBS) and 1% Zell Shield (Minerva Biolabs, Berlin, Germany). Grown cells wer8e transfected using the Fugene HD transfection reagent (Promega, Madison, WI, USA) according to the manufacturer's instructions. Calcium imaging experiments were performed 24 h after the transfection.

Figure S1

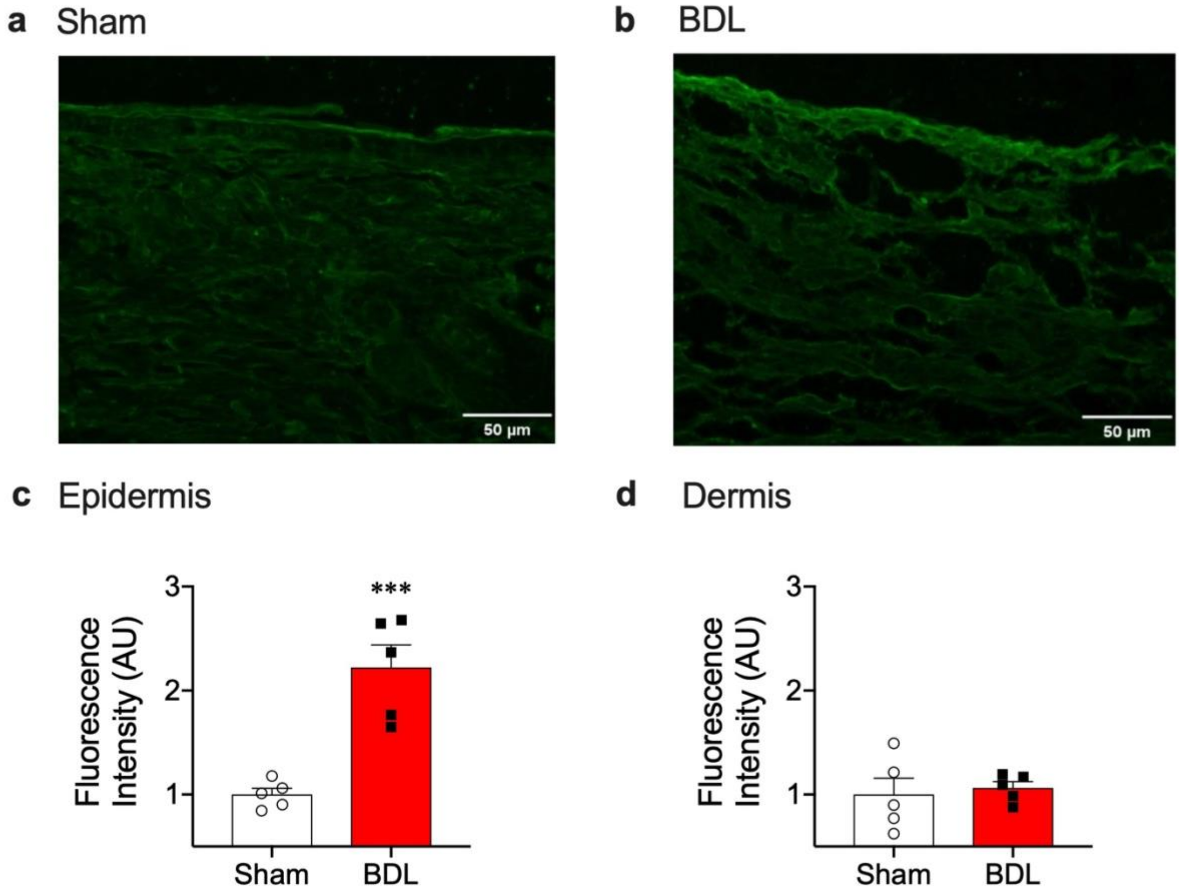

Figure S1. BAM22P is increased in the epidermal area of BDL mice. Representative images of the immunofluorescence analysis with BAM22P antibody in the skin of sham (a) and BDL (b). Summary of the fluorescence intensities in the epidermal (c) and dermal (d) regions of the sham and BDL from 5 separate samples. *** $p < 0.001$ (Student's *t*-test). Results are presented as means ± SEM

Figure S2

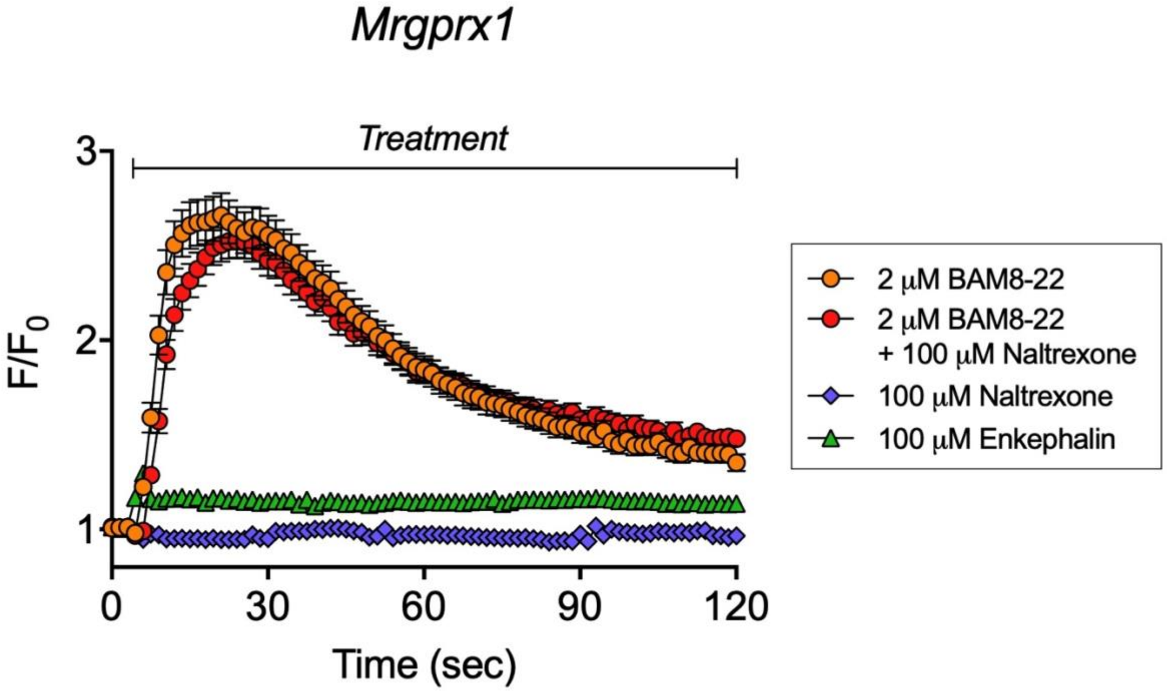

Figure S2. Naltrexone nor enkephalin do not have impact on MRGPRX1. The increase of intracellular calcium level by 2 μM BAM8-22 was not inhibited by μ-opioid antagonist naltrexone pretreatment on HEK293T cells transfected with *Mrgprx1*. Moreover, treatment of enkephalin did not induce noticeable intracellular calcium level increase.

Table S1. Human primer sequences for quantitative reverse transcription-polymerase chain reaction.

Target genes	Primer direction	Sequence
GAPDH	Forward	5'-GAA GGT GGA GGT CGG AGT C-3'
	Reverse	5'-GAA GAT GGT GAT GGG ATT TC-3'
PENK	Forward	5'-ATC CTC GCC AAG CGG TAT G-3'
	Reverse	5'-GGT TGT CCC CTG TTT CCA GA-3'
PCSK1	Forward	5'-GGA CCT CTG AGT ATG ACC CG-3'
	Reverse	5'-AGC TTT GGC ATT TAG CAA GCC-3'
PCSK2	Forward	5'-CTC TCA GGC ACG GTT TTC CA-3'
	Reverse	5'-ATC CTC GCC AAG CGG TAT G-3'

Table S2. Mouse primer sequences for quantitative reverse transcription-polymerase chain reaction.

Target genes	Primer direction	Sequence
Gapdh	Forward<br>Reverse	5'-AGG TCG GTG TGA ACG CAT TT-3'<br>5'-TGT AGA CCA TGT AGT TGA GG-3'
Penk	Forward<br>Reverse	5'-GAC AGC AGC AAA CAG GAT GA-3'<br>5'-GCA TAA AGC CCC CGT ATC TT-3'
Pcsk1	Forward<br>Reverse	5'-CGT TCA GTT CAA AAA GAC TCA GC-3'<br>5'-ACT CCT TTG CCA GTA ATA CCC T-3'
Pcsk2	Forward<br>Reverse	5'-GTG TGA TGG TTT TTG CGT CTG-3'<br>5'-GGG AGC TTT CGG ACT CCA A-3'
Mrgprx1	Forward<br>Reverse	5'-TCT CAT CCC ACG ACA CAG-3'<br>5'-AGC CAG AGT ACA ATG GTG TTT-3'
Mrgpra3	Forward<br>Reverse	5'-CTC AAG TTT ACC CTA CCC AAA-3'<br>5'-CCG CAG AAA TAA CCA TCC AGA-3'
Trpv1	Forward<br>Reverse	5'-CCA CTG GTG TTG AGA CGC C-3'<br>5'-TCT GGG TCT TTG AAC TCG-3'
Trpa1	Forward<br>Reverse	5'-GTC CAG GGC GTT GTC TAT CG-3'<br>5'-AGC ACT TCA CAC GAA GAA-3'
Gpbar1	Forward<br>Reverse	5'- CCT GGA ACT CTG TTA TCG CTC A-3'<br>5'-GCA CTC GTA GAC ACC TTT GGG-3'
